# Diversity and potential plant growth promoting capacity of seed endophytic bacteria of the holoparasite *Cistanche phelypaea* (Orobanchaceae)

**DOI:** 10.1038/s41598-023-38899-9

**Published:** 2023-07-22

**Authors:** Kristine Petrosyan, Sofie Thijs, Renata Piwowarczyk, Karolina Ruraż, Wiesław Kaca, Jaco Vangronsveld

**Affiliations:** 1 Department of Microbiology, Institute of Biology, Jan Kochanowski University, Uniwersytecka 7, 25-406 Kielce, Poland; 2 Environmental Biology Research Group, Centre for Environmental Sciences, Hasselt University, Agoralaan Building D, 3590 Diepenbeek, Belgium; 3 Department of Environmental Biology, Center for Research and Conservation of Biodiversity, Institute of Biology, Jan Kochanowski University, Uniwersytecka 7, 25-406 Kielce, Poland; 4 Department of Plant Physiology and Biophysics, Faculty of Biology and Biotechnology, Institute of Biological Sciences, Maria Curie-Skłodowska University, Akademicka, 19, 20-033 Lublin, Poland

**Keywords:** Microbiology, Plant symbiosis

## Abstract

Salt marshes are highly dynamic, biologically diverse ecosystems with a broad range of ecological functions. We investigated the endophytic bacterial community of surface sterilized seeds of the holoparasitic *Cistanche phelypaea* growing in coastal salt marshes of the Iberian Peninsula in Portugal. *C. phelypaea* is the only representative of the genus *Cistanche* that was reported in such habitat. Using high-throughput sequencing methods, 23 bacterial phyla and 263 different OTUs on genus level were found. Bacterial strains belonging to phyla Proteobacteria and Actinobacteriota were dominating. Also some newly classified or undiscovered bacterial phyla, unclassified and unexplored taxonomic groups, symbiotic Archaea groups inhabited the *C. phelypaea* seeds. γ-Proteobacteria was the most diverse phylogenetic group. Sixty-three bacterial strains belonging to Bacilli, Actinomycetes, α-, γ- and β-Proteobacteria and unclassified bacteria were isolated. We also investigated the in vitro PGP traits and salt tolerance of the isolates. Among the Actinobacteria, *Micromonospora* spp. showed the most promising endophytes in the seeds. Taken together, the results indicated that the seeds were inhabited by halotolerant bacterial strains that may play a role in mitigating the adverse effects of salt stress on the host plant. In future research, these bacteria should be assessed as potential sources of novel and unique bioactive compounds or as novel bacterial species.

## Introduction

Due to their high biological productivity, contribution to global greenhouse gas emissions and involvement in nutrient cycling, wetlands are considered as highly valuable ecosystems. Salt marshes are unique wetland systems characterized by harsh environmental conditions including periodic flooding and high salinity levels, nutrient levels, herbivore densities and also sea level rise. They harbor a halophilic vegetation^1,2^. These ecosystems are characterized by low numbers of dominating macrophytes, with predictable acidification and high salinity in the environment^3^. The plants growing in coastal salt marshes are an underexplored source of plant associated bacteria having a great potential to antimicrobial, enzymatic, plant growth promoting (PGP) and biodegrading traits^3^. In such salt marshes, the holoparasitic *Cistanche phelypaea* (Orobanchaceae) has our particular interest. The genus *Cistanche* includes about 25 species, and is found mainly in arid, semi-arid and halophytic habitats across Eurasia and North Africa^4^. Most species belonging to this genus require special environmental conditions for growth, i.e. extreme arid climate, poor and impoverished soils, large temperature fluctuations, intensive sunshine and low annual precipitation^5^. The obligate root parasite *C. phelypaea* has a limited area of distribution and is endemic to salt marshes at the Atlantic coast of Portugal and Spain^6–8^.

During evolution obligate root holoparasitic Orobanchaceae were subject to multiple modifications, e.g. loss of the roots and plastids^9,10^, and the production of large numbers of extremely small seeds which remain viable in the soil for many years^11,12^. The main storage material in the seeds of holoparasitic Orobanchaceae are lipids^13^. The crucial stages of the lifecycle of holoparasitic plants are seed germination, reaching the desirable host, gaining access to nutrients, and development of the new generation^14^. When host signals are received, seeds start germinating. Subsequently, the germinating seeds develop specialized organs called haustoria, that penetrate the host vascular system. Through such haustoria holoparasitic seeds can reach the water and nutrients of the host^15,16^.

In host-parasite interactions, the extreme conditions of salt marshes are challenging for both partners. *C. phelypaea* is parasitizing on roots of halophytic plants^6^. Thus, the holoparasite should possess defense mechanisms against the abiotic conditions in the saline soil, the high osmotic pressure of the soil solution, and must be able to cope with the salt tolerant nature of the host plant as well^17^. The parasitic plants are affected by salinity stress both directly and undirectedly. Although, the root parasites have very limited soil contact, they are indirectly affected by salt stress through the metabolism of their host. In natural habitats, most of the *Cistanche* species prefer their hosts growing under stress conditions, due to the high contents of nutrients that hosts accumulate in such habitats^18^. Salt stress inhibits the germination of *C. phelypaea* seeds directly and through changes in host signaling that strongly depends on the concentration of germination stimulants. The parasite response mechanisms to salinity are still unclear^19^. It was found that some Orobanchaceae accumulate high amounts of polyols^20^. The half-parasite *Viscum album* (mistletoe), parasitizing in trees, was reported to actively absorb polyols from the host and develop a host-specific polyol profile^21^. At higher salt concentrations, *Cuscuta campestris,* parasitizing on *Arabidopsis,* accumulated l-proline and decreased in this way the salt concentrations in the host compared to non-infected plants^22^.

Plant associated bacteria have a key role in plant fitness. They may survive in harsh abiotic conditions and stimulate the host metabolism in response to abiotic stresses^23^. Like their host plants, these microorganisms are strongly influenced by the salinity of the environment^24^. It was suggested that, in harsh habitats, the endophytes colonizing plant seeds have survival strategies that are different from endophytes of plants growing on less demanding soils and that they might be able to survive in seeds for a long period of time^25,26^. Endophytic bacteria often possess PGP traits like e.g*.* auxin production^27^. It has been proven that vegetation type, plant species, carbon and nutrient availability, soil type and structure, abiotic parameters of soil, particularly pH, as well as soil moisture have a direct impact on the diversity of microorganisms colonizing the roots^28,29^. These bacteria are transferred from the roots to other parts of the plant and reach the seeds that function as a vector from one generation to the next^30,31^. As many authors mentioned, the seeds of plants, including the holoparasitic *C. armena*, can harbor diverse endophytic bacterial communities adapted to survive in harsh abiotic conditions^23,26,32^. The most unique properties of the seed-associated bacterial endophytes are their vertical transmission and preservation in seeds for a long period of time^30,33,34^. Those are important capacities for the assemblage and establishment of the endomicrobiome of consecutive seed generations that consistently show similar endophytic communities with prominent dominating groups^34^.

Although, environmental and anthropogenic factors, such as oil spills, storms or high levels of eutrophication are challenging for organisms in natural salt marshes, the bacterial communities are generally quite resistant to extensive compositional changes^35^. On the other hand, differences between the composition of the microbial communities in the different zones of salt marshes have been reported^2^. Dominating plant populations, soil organic matter, phosphorous and nitrogen concentrations were observed to influence the succession of the bacterial communities^36^. Considering that the root parasite is fully dependent on its host plant, the microbial diversity of the holoparasite also depends on its host species and individual plant characteristics^37^.

The plant associated endophytic microbial diversity has been particularly well-studied in terrestrial ecosystems. However, the diversity and biological activity of wetland microbial communities is still highly underexplored and is often focusing on wastewater treatment^1,38^. From the endosphere of mangrove propagules^23^ and plant species growing in saline wetland ecosystems, various bacterial phyla have been isolated^39,40^. The four main bacterial phyla Proteobacteria, Firmicutes, Actinobacteria, and Bacteroidetes were identified as dominant phyla in both, terrestrial and aquatic ecosystems^23,28,38,41^. These bacterial phyla and several genera like *Acinetobacter*, *Bacillus*, *Micrococcus*, *Rhizobium*, *Staphylococcus* are often associated with seeds across a wide range of plant taxa^33,42–44^. These endophytes possess a considerable tolerance to a wide range of abiotic stresses typical for coastal environments, indicating their adaptive ability to this particular habitat^23^.

Special and extreme environments are rich in rare Actinobacteria and novel species. Rare Actinobacteria are usually difficult to isolate mainly due to their particular growth requirements and/or unknown culture conditions^45^. Actinobacteria from wetland ecosystems have a special interest. They produce structurally diverse bioactive components, like enzymes, antibiotics, antitumor and immune regulatory agents^45–47^. They can also play an important role as symbionts in plant-associated microbial communities^48^. A large diversity of endophytic Actinobacteria was identified in several plants growing in mangrove and salt marshes in various locations in India (Bhitarkanika region) and China (Shankou Mangrove Nature Reserve, Zhanjiang Mangrove Forest National Nature Reserve, Beilum Estuary National Nature Reserve). The most abundant endophytes in wetland ecosystems belong to the *Streptomyces*, *Nocardiopsis*, *Pseudonocardia*, *Saccharopolyspora, Agrococcus*, and *Micromonospora*^49–52^. However, isolation, diversity and biological activity of endophytic Actinobacteria from coastal salt marsh ecosystems still is not intensively explored, even though such environments seem to harbor unique and phylogenetically diverse endophytes^53^.

To date, there exist only a few studies concerning seed endophytic bacterial communities of holoparasitic plant seeds^32,54,55^. The endophytic bacterial community (culturable and unculturable) in ripe seeds of *C. phelypaea* growing in coastal salt marshes has not yet been investigated. There exist a limited number of studies on the biology and ecology of *C. phelypaea*. Most works concern the distribution, biomass, host range, the allelopathic potential, and the ecophysiological interactions between *C. phelypaea* and its host^6,7,17,56–58^.

The aim of our study was to investigate the diversity of the endophytic bacterial community of surface sterilized seeds of *C. phelypaea* growing in Iberian coastal salt marshes. We also studied the in vitro PGP traits of the bacterial isolates, such as production of indole, organic acids and siderophores and the ACC deaminase activity.

## Results

### Seed endophytic bacterial community composition

We used high-throughput sequencing methods to investigate the diversity and composition of the bacterial endophytic community from seeds of *C. phelypaea*. Alpha diversity indices (Shannon–Wiener biodiversity index, Chao1 and Simpson indexes) were 3.12, 165, 0.868 respectively with P value 0.05. A total of 263 different Operational Taxonomic Unit (OTU)s at genus level were found from 23 phyla (> 0.1%). Unclassified and unexplored taxonomic groups were found as well.

**Figure 1:**
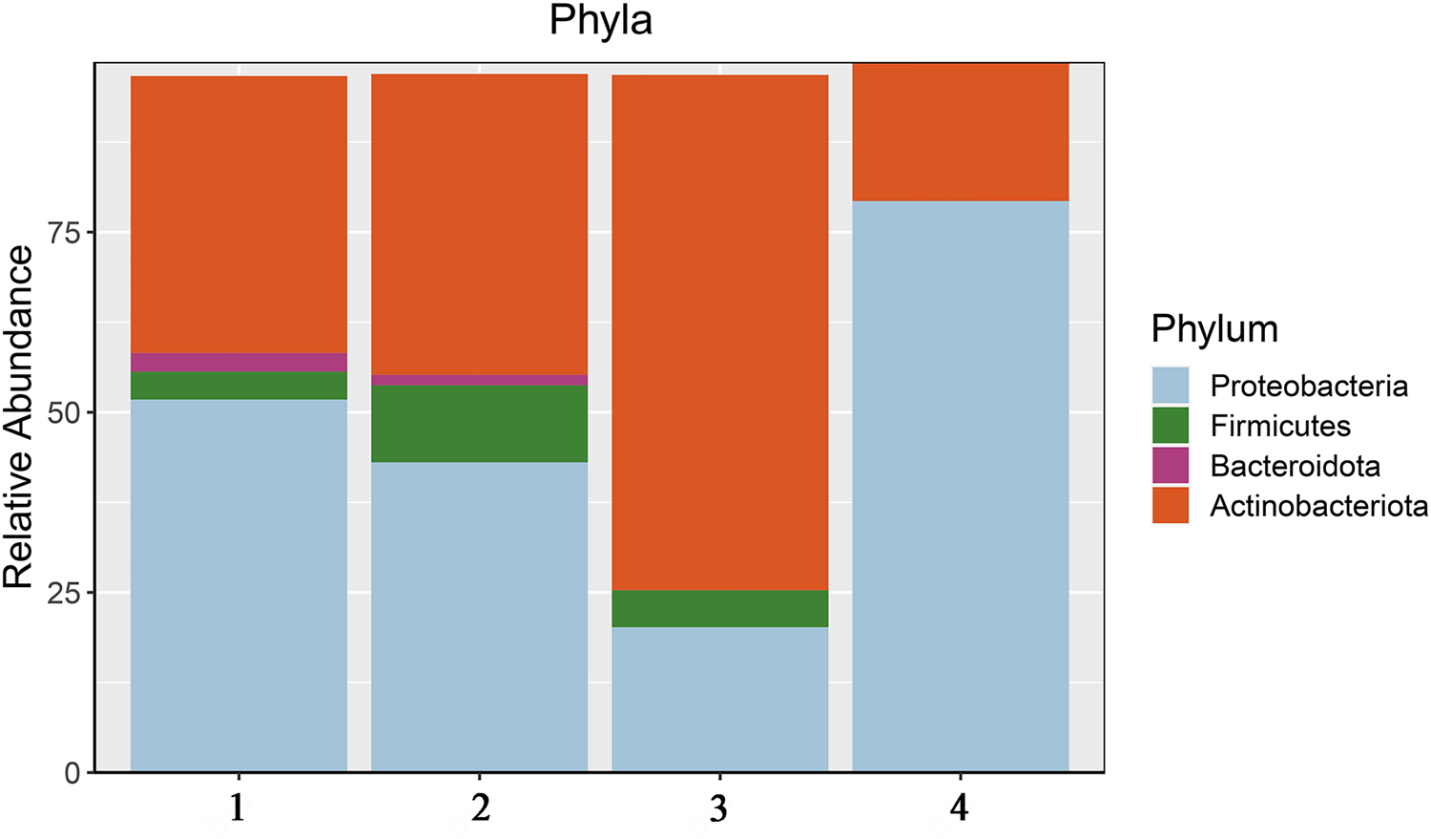
Relative abundance (> 1%) of dominating phyla of endophytic bacteria in four DNA replicates (1, 2, 3, 4) of *Cistanche phelypaea* seeds.

Proteobacteria (48.8%) and Actinobacteriota (43.0%) were the predominating phyla in four replicates. Bacteroidetes (1.2%) and Firmicutes (5.2%) were observed only in two and three out of the four replicates respectively (Fig. 1). Other phyla represented less than 1%. Besides well-known bacterial groups, the seeds were also colonized by newly classified or unknown ones. Among those was the recently proposed bacterial candidate phylum Entotheonellaeota. The other special phyla detected in the *C. phelypaea* seeds were the newly defined superphylum Patescibacteria^59^, and the phyla Armatimonadetes^60^ and Desulfobacteriota proposed by phyl. nov.^61^. The *C. phelypaea* seeds were also inhabited by symbiotic Archaea belonging to the Nanoarchaeota, Halobacterota and Crenarchaeota.

From the three classes of Proteobacteria, the γ-Proteobacteria were the most diverse compared to α- and β-Proteobacteria. Unclassified Proteobacteria at different taxonomic levels were found as well. Within the α-Proteobacteria, the orders Sphingomonadales and Rhizobiales were found in much higher abundances. At the order level γ-Proteobacteria was dominated by Enterobacterales and Pseudomonadales, and β-Proteobacteria were represented by Burkholderiales and Neisseriales (Table 1). The phylum Actinobacteriota was mainly represented by the orders Propionibacteriales with *Cutibacterium* at genus level, and Micromonosporales with the genera *Micromonospora* and *Microbacterium.* Besides well-known Actinobacteria, about 8.7% rare Actinobacteria^62^ were found: *Cryptosporangium*, *Actinokineospora*, *Pseudonocardia*, *Actinoplanes*, *Kineosporia*, and *Nocardia*. Finally, the third dominating phylum identified in the examined seeds were the Firmicutes with as most important orders the Lactobacillales and the Staphylococcales, with the genera *Streptococcus* and *Staphylococcus* respectively. From the order Bacillales, the families Bacillaceae and Planococcaceae were represented. More detailed information about dominating endophytic bacteria at different taxonomic levels is presented in Table 1.

**Table 1:** Cumulative list of dominating endophytic bacteria in the seeds of *Cistanche phelypaea* and their taxonomic information.

Phyla	Classes	Orders	Families	Genera*
Actinobacteriota	Actinobacteria	Micromonosporale	Microbacteriaceae	*Micromonospora*
				*Microbacterium*
		Propionibacteriales	Propionibacteriaceae	*Cutibacterium*
Proteobacteria	α-Proteobacteria	Sphingomonadales	Sphingomonadaceae	*Sphingomonas*
		Rhizobiales	Rhizobiaceae	*Allorhizobium-Neorhizobium-Pararhizobium-Rhizobium; NA*
	β-Proteobacteria	Burkholderiales	Burkholderiaceae	*Ralstonia*
		Neisseriales	Neisseriaceae	*NA*
	γ-Proteobacteria	Enterobacterales	Morganellaceae	*Proteus*
			Aeromonadaceae	*Aeromonas*
			Shewanellaceae	*Shewanella*
		Pseudomonadales	Pseudomonadaceae	*Pseudomonas*
				*NA*
			Moraxellaceae	*Acinetobacter*
				*Enhydrobacter*
Firmicutes	Bacilli	Lactobacillales	Streptococcaceae	*Streptococcus*
			Aerococcaceae	*Abiotrophia*
		Staphylococcales	Staphylococcaceae	*Staphylococcus*
		Bacillales	Bacillaceae	*Bacillus*
			Planococcaceae	*NA*
Unclassified groups at high taxonomic levels	NA	NA	NA	NA

Only taxonomic groups with an abundance > 10% are listed.

*Other genera were represented at abundances lower than 10% of the total seed endophytic community.

### Diversity and in vitro characterization of PGP bacteria isolated from the surface-sterile seeds

In order to obtain as complete as possible overview of the culturable members of the endophytic community of *C. phelypaea* seeds, different culture media were chosen. In total 63 bacterial strains belonging to Firmicutes (66.7%), Actinobacteriota (15.9%), α-, γ- and β-Proteobacteria (14.3%) and unclassified bacteria (3.2%) were picked up from the 1/869 rich, Flour1, PDA, R2A, 284, and YEDC media and subsequently identified (Table 2). The morphological characteristics of bacterial colonies and more detailed taxonomic information about isolated bacterial strains were presented in Supplementary Table S1 and Supplementary Fig. S1.

**Table 2:** Cumulative list of isolated culturable endophytic bacteria in the seeds of *Cistanche phelypaea* and their taxonomic information.

Count of isolated bacteria %	Phyla	Classes	Orders	Genera
66.7	Firmicutes	Bacilli	Bacillales	*Bacillus*
				*Paenibacillus*
				*Peribacillus*
15.9	Actinobacteria	Actinomycetia	Micromonosporales	*Micromonospora*
			Micrococcales	*Brevibacterium*
				*Plantibacter*
				*Janibacter*
3.2	Proteobacteria	α-Proteobacteria	Sphingomonadales	*Sphingomonas*
			Hyphomicrobiales	*Microvirga*
		β-Proteobacteria	Burkholderiales	*Ralstonia*
9.5				
1.6		γ-Proteobacteria	Xanthomonadales	*Stenotrophomonas*
3.2	Unclassified	Unclassified	Unclassified	Unclassified

**Figure 2:**
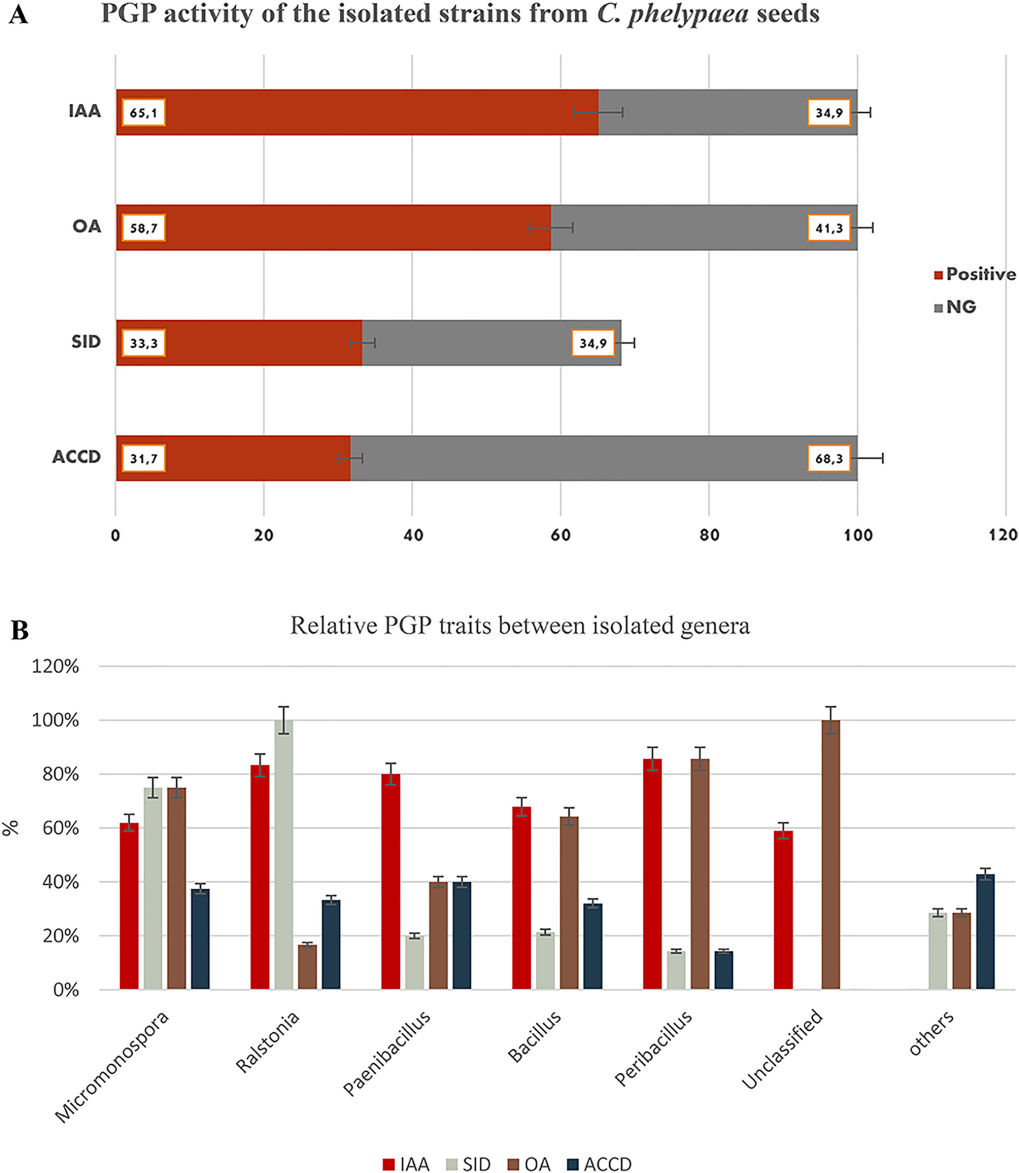
PGP activity of the strains isolated from the *Cistanche phelypaea* seeds. The figure (**A**) shows the IAA, ACCD, siderophores and organic acids’ production ability. positive-red, negative-gray. (**B**) Figure shows the IAA (red), ACCD (blue), siderophores (gray) and organic acids (light brown).

The isolated strains were also tested in vitro for various Plant Growth Promoting (PGP) properties which are presented in Fig. 2. Respectively, 41 (65.1%) and 37 (58.7%) of all isolated strains tested positive for the production of indole (IAA) and organic acids (OA) (Fig. 2a); among those the strains belonging to the genera *Ralstonia*, *Paenibacillus*, *Peribacillus* demonstrated the highest IAA production (Fig. 2b). Meanwhile, the strains belonging to *Micromonospora*, *Peribacillus* and unclassified strains demonstrated more positive responses for the production of organic acids (Fig. 2b).

For both, ACC-deaminase and siderophore production, respectively 20 (31.7%) and 21 (33.3%) of the investigated strains tested positive (Fig. 2a); among those 75% of the *Micromonospora* and 100% of the *Ralstonia* were positive for production of siderophores. In case of ACC deaminase, most positive responses were found for strains belonging to *Micromonospora*, *Ralstonia* and *Paenibacillus* (Fig. 2b). Among the β-Proteobacteria, *Ralstonia* spp. demonstrated the highest number of positive responses for siderophore production. Among the Actinobacteria, *Micromonospora* spp. demonstrated equally high positive responses for all tested PGP traits (Fig. 2b). Detailed information about the in vitro PGP traits is presented in Supplementary Table S1 and Supplementary Fig. S1.

### Salt tolerance of cultivated bacteria

In order to confirm the salt tolerance of the isolated strains the modified Brain–heart infusion (BHI) broth^63^ was used. After 7–10 days observation all tested bacterial strains demonstrated salt tolerance (Supplementary Fig. S1).

## Discussion

Salt marshes are natural habitats for many unique salt-tolerant plants^64^. Among these, the holoparasitic *C. phelypaea* can be highlighted. The heterotrophic lifestyle of parasitic plants was leading to several morphological, physiological and molecular adaptations with unique properties that make them an exciting group for study; the production of large numbers of very small seeds and the very specific conditions of germination are examples. Besides, they have significant effects on the ecosystems in which they occur^15^. The importance of wetlands, particularly salt marshes and the limited knowledge concerning the microbiology of such systems form the incentive for the current paper, focusing on the seed-associated endophytic bacterial community of the obligate root parasite *C. phelypaea* that is parasitizing on the roots of halophytic plant species^6^.

In a recent study^32^ we examined the bacterial seed endomicrobiome of another species from the genus *Cistanche*, *C. armena,* an endemic species growing in saline and arid habitats in Armenia. The seed endophytic bacterial community of *C. armena* is less diverse compared to that of *C. phelypaea* and consists mainly of spore forming, halophytic bacteria, with the Firmicutes as the predominating phylum (60.4%) (Table 3). From the seeds of *C. armena,* 10 bacterial phyla and 256 genera were identified. The structure and diversity of this endophytic bacterial community is related to the natural habitat of their host plant^32^.

**Table 3:** Comparison of dominant bacterial phyla (> 1%) in seeds of *Cistanche armena* and *C. phelypaea*.

	Proteobacteria (%)	Firmicutes (%)	Actinobacteriota (%)	Bacterioitoda (%)
*C. armena*^32^	32.9	**60.5**	6.5	1.0
*C. phelypaea*	**48.8**	5.2	**42.8**	1.2

The predominating phyla are indicated in bold.

In the present study we describe the structure and diversity of the endophytic bacterial community of seeds from an endemic *C. phelypaea* population growing in coastal salt marshes in Portugal. Proteobacteria, Actinobacteriota, Firmicutes and Bacteroidetes are well represented (> 1%) in surface sterile seeds (Fig. 1). Corresponding results were reported for the seeds of other holoparasitic Orobanchaceae species, *C. armena*^32^ and *Phelypanche ramosa*^54^.

Besides commonly known bacterial groups, the seeds *C. phelypaea* are also colonized by newly classified or unexplored bacterial phyla that have not been reported in such seeds before. Among these are Entotheonellaeota which, until now, were found mainly associated with marine sponges. Bacteria isolated from the marine sponge *Theonella swinhoei Y* are uncultured filamentous bacteria “Candidatus Entotheonella factor” and “Candidatus Entotheonella gemina”. Whole genome sequencing of *Ca. Entotheonella* demonstrated an extraordinarily rich genomic potential for the production of bioactive natural products with unique structures, uncommon biosynthetic enzymology and largely unknown properties^65^. Another interesting newly defined superphylum Patescibacteria has been found in groundwater, sediments, lakes, and other aquatic environments and was found associated with plants as well^59,66^. The role of Patescibacteria in plants is still unclear, although they have been discovered inside of tissues of different plants, in plant associated soils and in degrading plant biomass^60,66^. The phylum Armatimonadetes is assumed to harbor strains that are widely involved in degradation of plant material, substances based on polysaccharides^60^.

With 48.8%, the phylum Proteobacteria is the most abundant one in the seeds of *C. phelypaea* Fig. 1, Table 3). In seeds of *C. armena* Firmicutes were dominating (60.4%), followed by Proteobacteria with 32.9% (Table 3). In seeds of *C. phelypaea,* among the Proteobacteria, the γ-Proteobacteria were more diverse compared to α- and β-Proteobacteria (Table 1). In comparison, in *C. armena* seeds were only abundance by γ-Proteobacteria with as dominating orders Xanthomonadales, Pseudomonadales and Enterobacterales^32^. The dominating γ-Proteobacteria in seeds of *C. phelypaea* were Pseudomonadales and Enterobacterales. At family level, Morganellaceae, Aeromonadaceae, Shewanellaceae, Pseudomonadaceae, Moraxellaceae were the most abundant (Table 1). Classes of Proteobacteria showed diverse distribution tendencies in different ecosystems. In sediments of marine wetland ecosystems, a wide distribution of γ-Proteobacteria has been reported, and most of them seemed involved in sulfur reduction^67^. However, a high abundance of α- and β-Proteobacteria appeared in freshwater sediments; this should be correlated with pH and nutrients^68^.

The second most dominating bacterial phylum in seeds of *C. phelypaea* was the Actinobacteriota, with a more or less similar abundance as the Proteobacteria (43.0% against 48.8%). This is different from the seeds of *C. armena* in which Actinobacteriota represented only 6.5% (Table 3). The abundance of this phylum in the examined seeds is not unexpected, taking into account the habitat in which *C. phelypaea* is growing. Actinobacteriota indeed were reported more often as the most characteristic phylum in wetland ecosystems^1,3,45,47,48^. Until today, the community structure, diversity, biological activities and mechanisms of environmental adaptation of Actinobacteria to special and extreme environments have been poorly studied compared to more moderate and terrestrial environments^69^. In seeds of *C. phelypaea,* the most abundant genus of the Actinobacteriota was *Micromonospora* (38.1%). The genera *Microbacterium* and *Curtobacterium* also were dominating genera in seeds of *C. armena* growing in a saline, extremely arid environment in Armenia^32^. It seems that *Curtobacterium* has an overall distribution and is present in both, wet and arid ecosystems. Many studies mentioned *Curtobacterium* being a pathogen of economically important plants^70^. Moreover, this genus is known to harbor a highly diverse genomic potential for the degradation of carbohydrates, particularly with regard to structural polysaccharides^71^.

The seeds of *C. phelypaea* are colonized by rare Actinobacteria as well: *Actinoplanes, Actinokineospora, Cryptosporangium, Kineosporia*, *Nocardia, Pseudonocardia*. These bacteria are relatively difficult to isolate and cultivate in vitro due to difficulties in mimicking the conditions of their natural habitats. Rare Actinobacteria were isolated from different environments: the deep ocean, deserts, mangroves, plants, caves, volcanic rocks, and stones, which illustrates their wide distribution in the biosphere^72^. As reported by several authors, such bacteria are a potential source of novel bioactive compounds. Thus, it is essential to enhance our knowledge about their diversity and distribution in the environment^46,60^.

In case of *C. armena* seeds, Firmicutes was the predominating phylum with a total abundance of 60.5% (Table 3). The isolated Firmicutes belonged to the genera *Paenibacillus* (28%), *Bacillus* (41.9%) and some other genera (11.8%)^32^. Using a range of different growth media allowed us to isolate and cultivate a quite high diversity of bacterial strains from surface sterilized seeds of *C. phelypaea* (Table 2). Even though the total abundance of Firmicutes in seeds of *C. phelypaea* was quite low (5.2%), a majority of the cultivable bacterial strains belonged to this phylum (66.7%), followed by Actinobacteriota (15.9%), α-, γ- and β-Proteobacteria (14.3%) and unclassified bacteria (3.2%) (Tables 2, 3).

The potential PGP traits and salt tolerance of 63 isolated bacterial strains were examined (Fig. 2). It is often claimed that such PGP traits, e.g. production of siderophores, ACC deaminase, salicylic acid, and phytohormones such as auxins, gibberellins, cytokinins, and abscisic acid are part of the mechanisms that assist plants to cope with abiotic stresses^73–75^.

**Figure 3:**
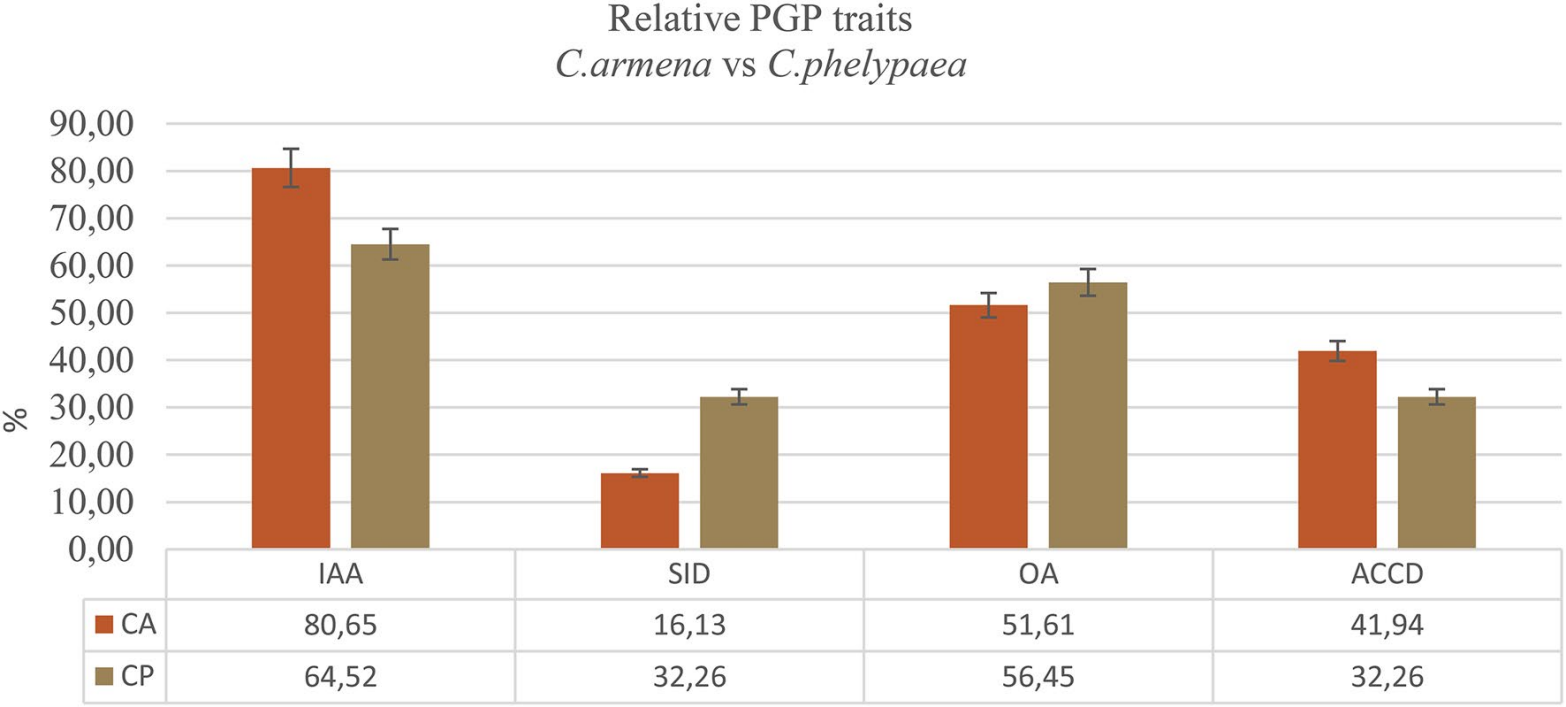
PGP traits of endophytic bacterial strains (%) isolated from the surface sterile seeds of *Cistanche armena* and *C. phelypaea*.

Based on in vitro tests, the selected bacterial isolates seem to possess several plant growth-promoting traits. 58.7% of the isolated strains belonging to various genera produced organic acids (OA), and also 65.1% showed able to produce IAA (Fig. 2a). Among those bacterial strains belonging to genera *Ralstonia* and *Peribacillus* showed the highest number of positive responses in the in vitro IAA production. In case of organic acids, the highest numbers of positive responses were found among *Micromonospora*, *Peribacillus* and Unclassified bacteria (Fig. 2b). Production of IAA and organic acids by many of the seed endophytes originating from coastal salt marshes (30–35% salt concentration) could be expected, since the seed endophytes isolated from *C. armena* from arid and saline habitats, also demonstrated high levels of IAA and organic acids production^32^. In the present study, 65.1% and 58.7% of isolates produce IAA and organic acids, respectively. Meanwhile, Petrosyan et al.^32^ demonstrated that most of bacterial strains isolated from the surface sterilized seeds of *C.armena* also could produce IAA (80.7%) and organic acids 51.6% (Fig. 3). Numerous studies have demonstrated that IAA-producing halotolerant bacteria increase the tolerance of plants to saline conditions. Organic acids production by rhizosphere microorganisms is one of the mechanisms to solubilize phosphorus (P) included in insoluble mineral compounds in soils^76^. Altogether, the seeds of both *Cistanche* species are inhabited by halotolerant bacteria that may have a role in mitigating the adverse effects of salt stress in the holoparasitic plant. However, it is still not clear if the PGP traits of these bacteria are beneficial only for the holoparasite either also for its host plant. It also remains unclear if the PGP capacities of seed endophytes may protect the embryo of the holoparasite against salt stress.

ACC deaminase capacity is another important trait of PGP endophytes. Many halophytic plants (*Salicornia europaea, Halimione portulacoides, Prosopis strombulifera* and *Limonium sinense*) are colonized by endophytes with ACC deaminase capacity^77^. ACC-deaminase can lower ethylene production under salt stress. Indeed, endophytes with ACC-deaminase capacity convert ACC (1-aminocyclopropane-1-carboxylic acid, the immediate precursor of ethylene in plants) into a-ketobutyrate and ammonia which helps the plants to reduce the negative effects of salinity stress on root development^77^. The major consequences of saline stress are nutrient imbalances and negative effects on plant physiology in general. Siderophores are high affinity iron-chelating compounds. Iron is a micronutrient, essential for plant growth and development, since it is an essential co-factor for a variety of reactions in plant cell metabolism and Fe-requiring enzymes. It is part of several metalloenzymes involved in photosynthesis and respiration. In saline soils, the bioavailability of Fe to plants is low. The role of microbial siderophores in Fe supply to plants and on the mitigation of saline stress in crop growth is well documented^78^. Respectively 33.3% and 31.7% of the isolated bacterial strains tested positive for siderophore and ACC deaminase production (Fig. 2a). Compared to *C. armena* that 16.1% were positive for siderophore and 42% scoped to positive for ACC deaminase (Fig. 3). Most positive responses were observed for Actinomycetia and β-Proteobacteria. It seems that β-Proteobacteria with *Ralstonia* spp. have a high siderophore producing capacity(Fig. 2b). *Sphingomonas* spp. (α-Proteobacteria) demonstrated the lowest level of positive responses for all in vitro tested PGP traits. Quite similar outcomes were observed for production of indole and organic acids. Also similar numbers of endophytes scored positive for ACC-deaminase and siderophore production (Fig. 2a). Among the Actinobacteria, *Micromonospora* spp. demonstrated the most positive responses for the in vitro tested PGP traits and thus might be considered as potentially the most favorable endophytes in *C. phelypaea* seeds. *Janibacter* strains, also belonging to the Micrococcales scored negative for all tested PGP assays (Supplementary Table S1).

## Conclusion

The holoparasitic *C. phelypaea,* which is parasitizing halophytic host plants, is the only representative of the genus *Cistanche* that is found in coastal salt marshes in Portugal. We explored the seed endophytic bacterial community of this holoparasite. We succeeded to isolate 63 bacterial strains, whilst using Illumina paired-end sequencing 263 different bacterial genera from 23 bacterial phyla were identified as seed endophytes of *C. phelypaea*. The 63 cultivable strains appeared to be salt tolerant and to possess plant growth-promoting traits like production of organic acids, IAA, siderophores and ACC deaminase. The genus *Micromonospora* harbored the most beneficial bacteria. These halotolerant bacteria have potential to mitigate the adverse effects of salt stress in the holoparasite. Possibly, they may also protect the embryo of the holoparasite against salt stress and maintain the seeds a long time viable. However, to confirm this, further studies concerning the beneficial effects of PGP seed endophytes on the holoparasite are required. In addition, the seeds of *C. phelypaea* represent and underexplored reservoir of diverse and novel endophytic Actinobacteria that may be of potential interest in the discovery of new bioactive compounds.

## Materials and methods

### Site description and seed sampling

**Figure 4:**
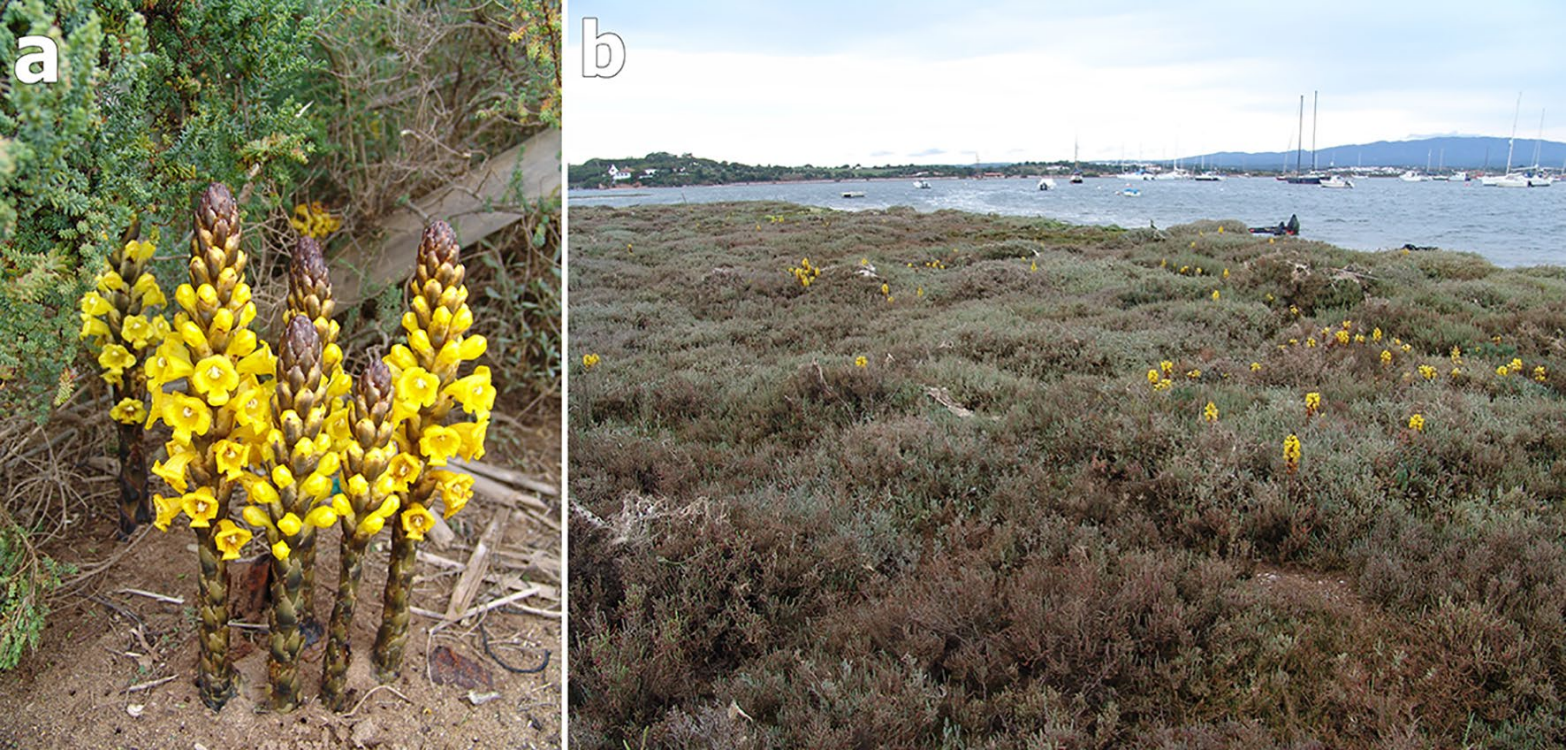
General habit of the studied species and its habitats: (**a**,**b**) *Cistanche phelypaea* in the coastal salt marshes in Portugal. Photos by R. Piwowarczyk.

The holoparasitic *Cistanche phelypaea* (L.) Cout. (Orobanchaceae) is found at the Atlantic coast of the Iberian Peninsula, in Portugal and Spain^7^. Ripe seeds were collected in sandy shores at the border of a salt marsh in Portugal, Algarve region, in the southwestern part of Alvor, in the estuary of the Rio de Alvor to the Atlantic Ocean, at about 10 m above sea level (37° 07′ 48.0′′ N, 8° 37′ 12.0′′ W). The Algarve coast is more sheltered with average high temperatures in summer around 25–28 °C and in winter minimal 11.5 °C. The average annual rainfall is about 500 mm. The area is an euhaline coastal lagoon with a salinity range from 30 to 35% and sandy-silty zones, periodically flooded and consists of coastal salt marshes^79^. A halophytic plant community is dominating including the *C. phelypaea* host species *Arthrocnemum macrostachyum* (Amaranthaceae*), Sarcocornia fruticose* (Amaranthaceae)*, Suaeda vera* (Amaranthaceae), and *Limoniastrum monopetalum* (Plumbaginaceae)^6^ (Fig. 4). The Iberian Peninsula is typified as a hotspot area of biodiversity with a high level of endemism^80^. The Ria de Alvor is actually a priority conservation area, is part of the European Ecological Network, Natura 2000 and is a RAMSAR wetland of international importance since 1996^64^.

Mature and dry seeds of *C. phelypaea* were collected from ripe fruits from at least 10 individual plants of the total population from the mentioned location in April 2012. Species were collected and identified by Renata Piwowarczyk. The herbarium materials were deposited in the Herbarium of the Jan Kochanowski University in Kielce (KTC), Poland. Vaucher specimens in the Herbarium KTC are not numbered yet. The herbarium materials are stored in a stable temperature of about 20 °C and a relative humidity of 30%. Field studies, the collection of plant and seeds material was complied with relevant local, institutional, national, and international guidelines, permissions, or legislation, and necessary permissions were obtained.

### Seed surface sterilization and homogenization

The aim of seed surface sterilization is to obtain only endophytic bacterial communities (culturable and unculturable). The surfaces of *Cistanche* seeds possess an alveolate ornamentation sculpture with polygonal and isodiametric cells with different sizes^81^. An efficient sterilization protocol is crucial. For this purpose, the seeds surface sterilization techniques described by Petrosyan et al.^32^ were used. The efficiency of the sterilization procedure was verified by plating 100 µl of the last rinsing water on 869 rich medium^82^. Subsequently, the surface sterilized seeds were mechanically homogenized in 0.5 ml 10 mM MgSO_4_ solution using a sterile pellet pestle (Kimble®). The homogenization process was accomplished using 5–6 metal stainless steel bead balls (2.8 mm) and a two-bladed mixer mill (Retsch MM400, Germany) for 15 min at 25 Hz. Part of the homogenous suspension was stored at − 80 °C for DNA extraction, another part was used for isolation of culturable bacteria (see below).

### Amplification of the 16S rRNA gene fragment and preparation of the next-generation sequencing library

For isolation and identification of the total (cultivable and uncultivable) bacterial community, the homogenized suspension of the surface sterilized seeds was used. The DNA isolation was performed in 4 replicates using the Mobio PowerPlant protocol based on the PowerPlant® Pro DNA Isolation Kit and patented solution (Inhibitor Removal Technology® IRT) for removal of PCR inhibitors from plant extracts during the isolation process.

All isolated DNA samples were subjected to bacterial 16S rRNA gene amplicon PCR. In the first round of 16S rRNA gene PCR, an amplicon of 291 bp was generated, using primers 515F-GTGYCAGCMGCCGCGGTAA and 806R- GGACTACNVGGGTWTCTAAT^83^, with an Illumina adapter overhang nucleotide sequence, resulting in the following sequences, 515F-adaptor: 5′-TCG TCG GCA GCG TCA GAT GTG TAT AAG AGA CAG-3′ and 806R-adaptor: 5′-GTC TCG TGG GCT CGG AGA TGT GTA TAA GAG ACA G-3′. For the first round of polymerase chain reactions (PCR) the Q5 High-Fidelity DNA Polymerase system (M0491, NEB) was used. 16S rRNA gene PCR were performed in 25 μl volumes containing 1 μl of extracted DNA, 1 × Q5 Reaction Buffer with 2 mM MgCl_2_, 200 μM dNTP mix, 1 × Q5 High GC Enhancer, 0.25 μM forward or reverse primer, 0.02 U µl^−1^ Q5 High-Fidelity DNA polymerase, 0.5 μl mitoPNA blocker (2 μM final concentration added from a 50 μM stock) and 0.5 μl plastidPNA blocker per sample^84^. Thermocycling conditions included an initial denaturation at 98 °C for 3 min, denaturation at 98 °C for 10 s, annealing at 56 °C for 30 s and extension at 72 °C for 30 s, all three steps were repeated for a total of 35 cycles and finally 7 min extension at 72 °C. The reaction was ended by cooling at 4 °C. The amplified DNA was purified using the AMPure XP beads (Beckman Coulter) and the MagMax magnetic particle processor (ThermoFisher, Leuven, Belgium). 5 μl of the cleaned PCR product was used for the second PCR attaching the Nextera indices. Indexing was performed using the Nextera XT™ library preparation kit ((Nextera XT Index Kit v2 Set A (FC-131-2001) and D (FC-131-2004), Illumina, Belgium). For these PCR reactions, 5 μl of the purified PCR product was used in a 25 μl reaction volume and prepared following the 16S Metagenomic Sequencing Library Preparation Guide. PCR conditions were the same as described above, but the number of cycles reduced to 20, and 55 °C as annealing temperature. PCR products were cleaned with the Agencourt AMPure XP kit, and then quantified in the Qubit 2.0 Fluorometer (Invitrogen). The libraries were pooled in equimolar concentrations to 4 nM using 10 mM Tris pH 8.5 prior to sequencing on the Illumina MiSeq. Samples were sequenced using the MiSeq Reagent Kit v3 (600 cycle) (MS-102-3003) and 15% PhiX Control v3 (FC-110-3001). For quality control, a DNA-extraction blank, PCR blank and ZymoBIOMICS Microbial Mock Community Standard (D6300) to test efficiency of DNA extraction (Zymo Research) were included throughout the process.

### Bioinformatic processing of reads

Sequences were demultiplexed using the Illumina Miseq software, and subsequently quality trimmed and primers removed using DADA2 1.10.1^85^ in R version 3.5.1. Parameters for length trimming were set to keep the first 290 bases of the forward read and 200 bases of the reverse read, maxN = 0, MaxEE = (2,5) and PhiX removal. Error rates were inferred, and the filtered reads were dereplicated and denoised using the DADA2 default parameters. After merging paired reads and removal of chimeras via the RemoveBimeraDenovo function, an amplicon sequence variant (ASV) table was built and taxonomy assigned using the SILVA v138 training set^86,87^. The resulting ASVs and taxonomy tables were combined with the metadata file into a phyloseq object (Phyloseq, version 1.26.1)^88^. Contaminants were removed from the dataset using the package Decontam (version 1.2.1) applying the prevalence method with a 0.5 threshold value^89^. A phylogenetic tree was constructed using a DECIPHER/Phangorn pipeline as described before^90^.

### Data visualization and statistical analyses

The ASV table was further processed removing organelles (chloroplast, mitochondria), and prevalence filtered using a 2% inclusion threshold (unsupervised filtering) as described by Callahan^85^. Alpha-diversity metrics such as Chao1, Simpson’s and Shannon’s diversity indexes were calculated on unfiltered data using scripts from the MicrobiomeSeq package. Hypothesis testing was done using analysis of variance (ANOVA) and the Tukey Honest Significant Differences method (Tukey HSD). When assumptions of normality and homoscedasticity were not met, a Kruskal–Wallis Rank Sum test and a Wilcoxon Rank Sum test was performed. The results were summarized in boxplots and relative abundances were calculated and visualized in bar charts using Phyloseq. All performed statistical tests were corrected for multiple testing and alpha < 0.05 was considered as statistically significant. All graphs and figures were generated in R version 4.0.4, Microsoft Office Excel 2010 software.

### Isolation and cultivation conditions of culturable endophytes

The first part of the obtained suspension (see above) was used for DNA extraction, the second part was for isolation of culturable bacteria. In order to cultivate as many as possible of the seed endophytes, 100 µL of the seed suspension was plated onto 1/869 rich^82^, Potato Dextrose Agar (PDA) (Sigma-Aldrich), R2A (Sigma-Aldrich), 284^91^, Flour-yeast extract-sucrose-casein hydrolysate agar (Flour1) and extract-casein hydrolysate agar (YECD)^92^. Besides the undiluted macerate, serial dilutions were prepared from 10^–1^ to 10^–3^ cfu ml^1^.

After inoculation, the Petri dishes with the different media were incubated at 30 °C for 7 days. The bacterial growth and diversity of colonies were evaluated for both undiluted and diluted seed suspensions. For further experiments, single, morphological distinct colonies were picked and purified. Subsequently, they were grown (in triplicate) in 96-well master blocks at 30 °C for 7 days shaken at 150 rpm. One block was used for DNA-extraction, the second one for PGP assays and the third was stored at − 45 °C in 15% glycerol (75 g glycerol, 4.25 g NaCl, 425 ml dH_2_O) solution.

### Genomic DNA extraction and taxonomic identification of the culturable endophytic bacterial strains

The DNA isolation was performed using standard procedure for DNA isolation from bacterial pellets with MagMAX Express 96 (APPLIED BIOSYSTEMS, Finland). DNA was quantified in a Qubit® 2.0 Fluorometer (Thermo Scientific, US) and a Nanodrop spectrophotometer (Thermo Scientific, US) with an A260/A280 ratio of 1.7–2.0 was used for checking the purity. The near full-length sequences of the 16S rRNA gene were amplified using 27f (5-AGAGTTTGATCMTGGCTCAG-3) and 1492r (5-GGTTACCTTGTTACGACTT-3) primers. A reaction volume of 25 μl per sample was containing 1 μl of DNA, 1 × Q5 Reaction Buffer (2 mM MgCl_2_), 0.25 μM 27f and 1492r primers, 200 μM dNTP mix and 0.02 U µl^−1^ Q5 High-Fidelity DNA polymerase and 1 × 5 × Q5 High GC Enhancer^84^. The 1/10 and 1/20 dilution for some DNA samples were done. PCR conditions were the same as described above, but the number of cycles reduced to 30, and 55 °C annealing temperature. The samples for 16S rRNA Sanger sequencing were shipped to Macrogen (https://www.macrogen-europe.com/). Sequences were quality filtered using Geneious v4.8 and were analyzed using the ribosomal database SILVA (https://www.arb-silva.de/aligner/). Bacteria were identified based on the PCR amplification of partial 16S rDNA gene sequences and annotated using the NCBI GenBank databases by the program Standard Nucleotide BLAST and RDP database (https://rdp.cme.msu.edu/seqmatch/seqmatch_intro.jsp).

### Plant growth promoting (PGP) traits

Considering the growth conditions of *C. phelypaea* the endophytic bacterial isolates were tested for their in vitro plant growth promoting traits and salt tolerance in high salt concentration (6.5%). All tests were performed at least two times.

The *tryptophanase activity* and *Indole-3-acetic acid (IAA) production* ability was tested using the Salkowski reagent^32^. The *organic acid production* was determined using the method of Cunningham and Kuiack^93^. *ACC-deaminase activity* was tested in SMN medium with 5 mM ACC^94^. *Production of siderophores* was assessed by using the 284 medium with 0.25 μl optimal iron concentration with CAS solution^95^. The detailed descriptions of methods were presented previously^32^.

### Salt tolerance

Salt tolerance ability of isolated bacteria was tested using the modified Brain–heart infusion (BHI) broth^63^ with composition: Peptic digest of animal tissue 10 g; Heart infusion 10 g; Glucose 1 g; Sodium chloride 65 g; Bromocresol purple 0,016 g per liter with pH 7.2. Bacteria were incubated for 5–7 days at 30 °C at 150 rpm agitation. After 7 days reaction time, the samples were observed for visible growth (turbidity) or color change. The bacterial growth (increase in turbidity) and/or change in the color of broth from purple to yellow was considered as positive. BHI without bacteria was chosen as negative control.

### Taxonomic classification of isolates and delineation of molecular OTUs

Ribosomal RNA gene sequences from bacterial isolates were compared with reference sequences from the GenBank databases, using Basic Local Alignment Search Tool (BLAST) software (http://blast.ncbi.nlm.nih.gov/Blast.cgi) and the Ribosomal Database Project (RDP) website (http://rdp.cme.msu.edu/), and isolates were assigned to species or the highest taxonomic rank possible.

## Supplementary Information

Supplementary Legends.

Supplementary Table S1.

Supplementary Figure S1.

## Data Availability

The sequence data available in the NCBI Genbank (https://www.ncbi.nlm.nih.gov/) Sequence Read Archive with BioSample accession number SAMN26931786. The partial 16S rRNA gene sequences received from of all studied strains were deposited in the GenBank database under the accession numbers presented in Supplementary Table S1.
