# Supplementary material for: Diversity and potential plant growth promoting capacity of seed endophytic bacteria of the holoparasite Cistanche phelypaea (Orobanchaceae)
Source: Sci Rep. 2023 Jul 22;13:11835. doi: 10.1038/s41598-023-38899-9 (PMC10363106; doi:10.1038/s41598-023-38899-9)
Supplement: Supplementary file 1 — Supplementary Legends. [file 41598_2023_38899_MOESM1_ESM.docx]

**Diversity and potential plant growth promoting capacity of seed endophytic bacteria of the holoparasite *Cistanche phelypaea* (Orobanchaceae)**

**Kristine Petrosyan, Sofie Thijs, Renata Piwowarczyk, Karolina Ruraż, Wiesław Kaca and Jaco Vangronsveld**

**Description of Supplementary Tables and Figures**

**Supplementary Table S1.** *In vitro* tests of the isolated endophytic bacterial strains of seeds of *Cistanche phelypaea*, their taxonomy and GenBank accession numbers (.xlsx).

**Supplementary Figure S1.** Bacterial morphological diversity of isolates and the results of the PGP assays and salt tolerance test (**a-**salt tolerance**, b-** ACC deaminase**, c-**IAA production**, d-**siderophores production) (JPG).
