# Supplementary figures and images for: Diversity and potential plant growth promoting capacity of seed endophytic bacteria of the holoparasite Cistanche phelypaea (Orobanchaceae)
Source: Sci Rep. 2023 Jul 22;13:11835. doi: 10.1038/s41598-023-38899-9 (PMC10363106; doi:10.1038/s41598-023-38899-9)

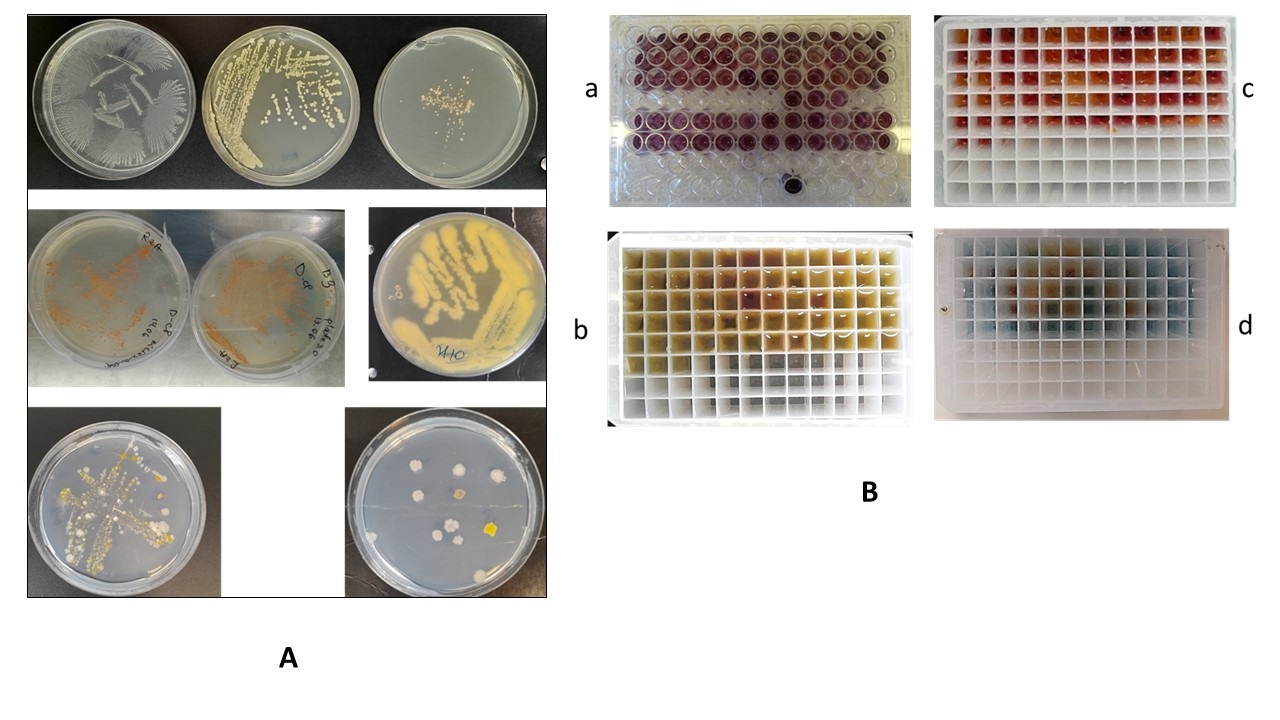

Supplement: Supplementary file 3 — Supplementary Figure S1. [file 41598_2023_38899_MOESM3_ESM.jpg]
